# Biomacromolecules, Biobased and Biodegradable Polymers: 2018–2019

**DOI:** 10.3390/polym13030453

**Published:** 2021-01-31

**Authors:** Seunho Jung

**Affiliations:** Department of Bioscience and Biotechnology, Department of Systems Biotechnology, Institute for Ubiquitous Information Technology and Applications (UBITA), Konkuk University, Seoul 05029, Korea; shjung@konkuk.ac.kr

This editorial introduces the most cited papers published in the years 2018–2019 in the section “Biomacromolecules, Biobased and Biodegradable Polymers” of the journal Polymers. They are mainly research review articles. The main topics appear related to biodegradable, biocompatiable, non-toxic biopolymers, polysaccharides, drug delivery, tissue engineering, hydrogels. The most used biopolymers are chitosan, cellulose, alginate and hyaluronic acids. Among 500 papers in this section, top 5% cited papers are discussed in this editorial.

The most cited paper is on the review for the cellulose aerogels [1]. Aerogels manufactured using cellulose, a renewable and biodegradable natural polymer, have various advantages such as regeneration, biocompatibility, biodegradability, low density, high porosity, and large specific surface area. Three types of aerogels made from natural cellulose aerogels (nano cellulose aerogels and bacterial cellulose aerogels), regenerated cellulose aerogels and cellulose derivatives have been applied in a variety of applications in adsorption and oil/water separation applications. Other cellulose-related papers included in the top 5% relate to emulsion formation and stabilization by cellulose [2] and injectable and gellable chitosan formulations filled with cellulose nanofibers [3]. The one introduced various aspects of the new role of modified cellulose as an emulsion stabilizer in various applications in the food, cosmetics, pharmaceutical, paint and construction industries as a flow modifier (thickener) or an emulsifier. The other paper, a viscous chitosan (CHI) solution filled with cellulose nanofiber (CNF) was proposed as non-cellularized injectable suspensions for viscosupplementation of the intervertebral disc nucleus pulposus tissue. Neutralization of a flowing weakly acidic CNF/CHI suspension resulted in a composite hydrogel in which nanofibers reinforced the CHI matrix. No leakage of injection solution through the syringe was observed and it showed that the gelled formulation could restore the disk height and recover the loss of mechanical properties typically associated with disk degeneration.

The second most cited paper is a review of “Chitosan Based Self-Assembled Nanoparticles in Drug Delivery” [4]. Chitosan, a cationic polysaccharide obtained through alkali deacetylation of chitin poly(*N*-acetylglucosamine), has excellent biological properties such as biocompatibility, biodegradability, mucosal adhesion, and non-toxicity, which is effective in improving biodistribution, increasing specificity and sensitivity. Because of this, chitosan can be a good candidate as a platform for developing drug delivery systems with reduced pharmacological toxicity. In particular, chitosan nanoparticles are known to be suitable for non-invasive drug administration routes such as oral, nasal, pulmonary, and ocular routes. In this review, the authors introduce a strategy applied to drug delivery by grafting a hydrophobic moiety that promotes self-assembly to chitosan to obtain chitosan nanoparticles or by forming polyelectrolyte complexes. Other papers on chitosan, which are included in the top 5%, include chitosan-modified nanoparticles for controlled release drug delivery [5], chitosan derivatives for introducing new functions [6], and 3D-printable alginate-chitosan polyion complex hydrogels [7]. In the first paper on drug delivery, chitosan (CS) was used to synthesize PLGA nanoparticles (NP), and the CS-PLGA nanoparticles were used to achieve sustained drug release and improved bioavailability. The authors have shown that CS-modified NP can be successfully used as a carrier for anticancer drugs (paclitaxel, PTX). The second paper on chitosan derivatives well summarized recent methods for the modification and derivatization of chitin and chitosan under various experimental conditions. In particular, it introduces recent methods and strategies for functionalization such as click chemistry approaches, grafting on copolymerization, binding to cyclodextrins, and reactions in ionic liquids. The third review paper on the alginate-chitosan polyion composite hydrogel describes how to prepare sophisticated scaffolds for tissue engineering using three-dimensional (3D) printing. In this paper, by dispersing chitosan powder in the alginate solution, the viscosity of the alginate solution was increased 1.5 to 4 times, so that it could be effectively used as a 3D printing material. It was also able to effectively attach human adipose-derived stem cells (hASC) to this 3D-printed hydrogel and showed its fine biocompatibility, demonstrating its potential to be used as a scaffold for tissue engineering. 

4D printing is a technology that uses traditional 3D printers to introduce product designs to smart materials and transforms them into the intended design using specific conditions or types of stimuli such as temperature, pressure, humidity, pH, wind or light. 4D printing can be viewed as a continuum of 3D printing technology that can print objects that change over time. One of the most cited papers is the review paper on polymeric materials for “4D Printing in Biomedical Applications” [8] where the current applicability, each activation method, characteristics and future prospects of various polymer materials used for 4D printing are introduced in detail.

In general, polysaccharides are unique scaffold materials capable of forming three-dimensional structures that support cell proliferation and regeneration processes due to their hydrophilicity, biocompatibility, biodegradability, abundance, and the presence of derivatizable functional groups. One of the interesting review papers titled as “Polysaccharide Based Scaffolds for Soft Tissue Engineering Applications” introduce the developmental and biological aspects and clinical studies of the skeleton possessed by four global polysaccharides: alginate, chitosan, hyaluronic acid and dextran [9]. In this paper, the authors describe customized scaffold fabrication methods that can effectively respond to changes in temperature, pH, or other physiological stimuli by inducing a specific spatial arrangement of the scaffold at the target site by designing an appropriate formulation through various modifications of polysaccharides.

The final paper I introduce here is on hyaluronic acid (HA), the most expensive natural biopolymer commercially. HA, a glycosaminoglycan composed of disaccharide units of *N*-acetyl-d-glucosamine and d-glucuronic acid, is involved in many important processes, including cell signaling, wound repair, tissue regeneration, morphogenesis, stromal tissue and pathology biology with various unique biochemical properties such as biocompatibility, biodegradability, mucosal adhesion, hygroscopicity and viscoelastic properties. In this review paper titled as “Hyaluronic Acid in the Third Millennium”, the overall characteristics of HA including physicochemical, structural and rheological properties were introduced with new methods recently developed for industrial production and chemical derivatization of HA [10].

It is worth mentioning that the above papers described in the section “Biomacromolecules, Biobased and Biodegradable Polymers” in 2018–2019 has been cited more than sixteen times.

## Data Availability

Not applicable.
